# Interactions of ABCG2 (BCRP) with epidermal growth factor receptor kinase inhibitors developed for molecular imaging

**DOI:** 10.3389/fphar.2014.00257

**Published:** 2014-11-21

**Authors:** Israa Qawasmi, Miriam Shmuel, Sara Eyal

**Affiliations:** Institute for Drug Research, School of Pharmacy, Faculty of Medicine, The Hebrew University of JerusalemJerusalem, Israel

**Keywords:** epidermal growth factor receptor, epidermal growth factor receptor kinase inhibitors, breast cancer resistance protein, *P*-glycoprotein, imaging, positron emission tomography

## Abstract

The objective of this study was to investigate *in vitro* the interactions between novel epidermal growth factor receptor kinase inhibitors (EGFRIs) developed for positron emission tomography (PET) imaging and the major eﬄux transporter breast cancer resistance protein (BCRP/ABCG2). Seven compounds were evaluated, using the ATPase activity assays and Madin-Darbey canine kidney (MDCK) cells overexpressing BCRP. Five of the tested compounds activated BCRP ATPase to various extent. Overexpression of BCRP conferred resistance to ML04, ML06, methoxy-Br-ML03, and PEG6-ML05 (IC_50_ values for inhibition of control cell proliferation 2.1 ± 0.6, 2.2 ± 0.7, 1.8 ± 1.2, and 2.8 ± 3.1 μM, respectively, compared to >50 μM in MDCK-BCRP cells). At submicromolar concentrations, none of the EGFRIs significantly inhibited BCRP. Immunoblotting studies indicated that BCRP expression is evident in cell lines utilized for *in vivo* tumor grafting in small animal PET imaging studies. Thus, the intensity of EGFRIs radioactivity signals previously observed in tumor xenografts reflects an interplay between transporter-mediated distribution of the probe into tumor cells and target binding. Concomitant use of eﬄux transporter inhibitors may help distinguish between the contribution of eﬄux transport and EGFR binding to the tissue signal.

## INTRODUCTION

Receptor tyrosine kinases play a key role in vital cellular functions, such as cell growth, differentiation, proliferation, and survival. Therefore, it is not surprising that enhanced activity of tyrosine kinases can lead to proliferative disease, including malignant tumors (Levitzki and Gazit, 1995; Levitzki and Mishani, 2006; Yarden and Pines, 2012). Accordingly, inhibition of hyperactive tyrosine kinase signaling pathways has emerged as a promising strategy for the treatment of cancer, and several TKIs have been approved by the FDA (Levitzki and Mishani, 2006; Mishani and Hagooly, 2009; Poot et al., 2013). Nevertheless, response rate is highly variable, in particular among patients treated with small-molecule TKIs directed against the EGFR (Poot et al., 2013).

The most established cause of secondary resistance is the occurrence of mutations in the catalytic domain of the kinase, which restrict the binding of currently available TKIs (Levitzki and Mishani, 2006). In addition, primary or secondary resistance may result from insufficient intracellular drug concentrations, due to poor uptake (Wang et al., 2008; Engler et al., 2011; Mandery et al., 2012) or active eﬄux transport (Ozvegy-Laczka et al., 2005; Eadie et al., 2014). Two prominent transporters involved in drug eﬄux from cells are *P*-gp (multidrug resistance protein 1) and the BCRP (ABCG2), members of the ABC family of membrane transporters (Szakács et al., 2006). *P*-gp and BCRP are expressed in tissues involved in drug absorption and elimination (intestine, liver, and kidney) and in barriers to drug distribution, such as the blood–brain barrier and the placenta. Hence, they play a key role in the pharmacokinetics of substrate drugs (Eyal et al., 2009). These transporters have also been implicated in multidrug resistance due to active removal of chemotherapeutic agents from tumor cells. *P*-gp and BCRP substrates include TKIs, although the relative affinities and the nature of interaction with ABC transporters vary among these compounds (Eadie et al., 2014). For example, gefitinib is a BCRP transported substrate and inhibitor at low concentrations (Ozvegy-Laczka et al., 2004), whereas nilotinib appears to be a BCRP transported substrate at submicromolar concentrations and an inhibitor at micromolar concentrations (Eadie et al., 2014).

Due to the variability in EGFRIs pharmacokinetics and patient response, PET imaging with radiolabeled EGFRIs has emerged as an important tool in the development of these compounds (Levitzki and Mishani, 2006; Mishani and Hagooly, 2009; Slobbe et al., 2012). Initial PET studies with fluorine-18-labeled reversible EGFR inhibitors in tumor-bearing animals demonstrated relatively low uptake or fast clearance of the reversible EGFRIs from the tumor area, at least in part due to active eﬄux from tumor cells (Mishani et al., 2008; Slobbe et al., 2012). Nevertheless, the interaction of gefitinib (Kawamura et al., 2009) and sorafenib (Asakawa et al., 2011) with *P*-gp and BCRP has become the basis for their evaluation as PET probes of the functional activity of these transporters at the blood-brain barrier, in which they restrict drug distribution into the brain (Eyal et al., 2009).

In order to enhance treatment and imaging efficiency, irreversible EGFR kinase inhibitors were developed on the basis of 4-(phenylamino) quinazoline and quinoline core structures (Mishani et al., 2004, 2005, 2008; Shaul et al., 2004; Abourbeh et al., 2007; Dissoki et al., 2007; Mishani and Hagooly, 2009). However, the tumor uptake of some of these compounds was modest, and one of them, the irreversible EGFRI ML04, was found to be a *P*-gp substrate (Abourbeh et al., 2007).

**FIGURE 1:**
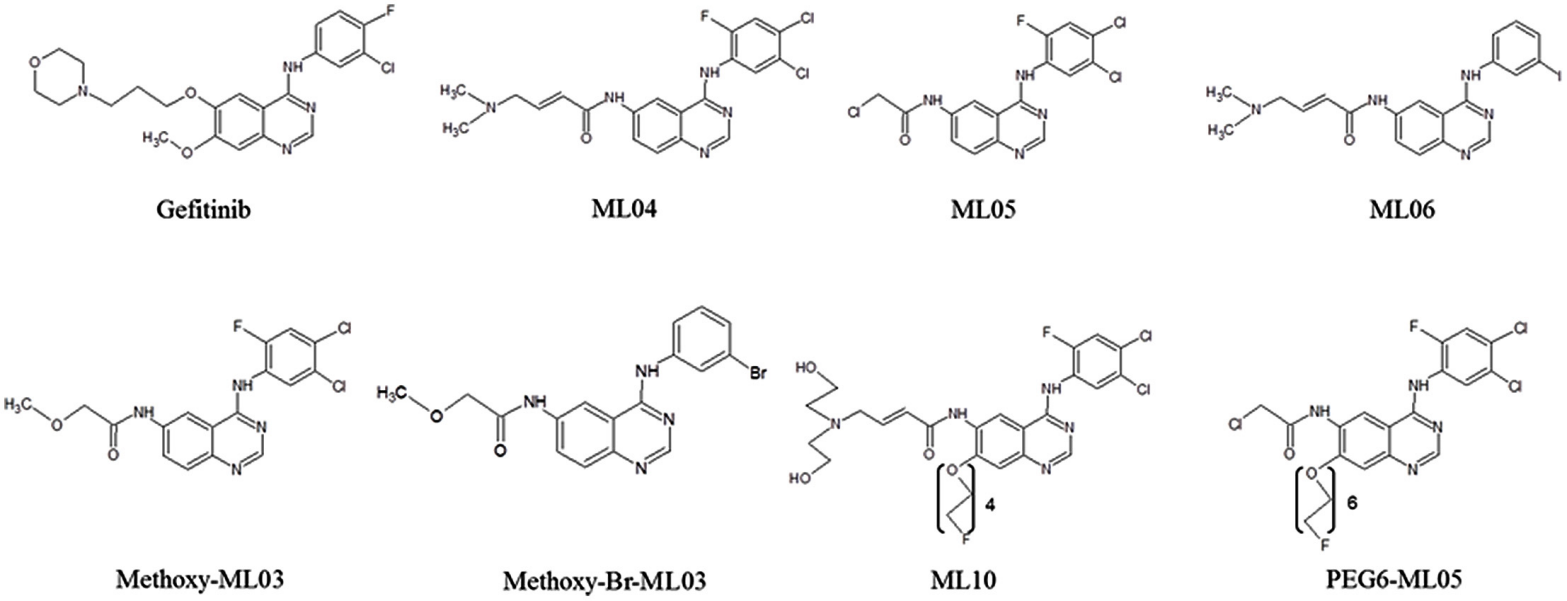
**Chemical structure of compounds investigated in this study**.

Here, we evaluated *in vitro* the interactions of seven novel EGFRI developed as PET bioprobes with BCRP, in order to better understand the factors that affect their biodistribution. The evaluated compounds were ML04 (Mishani et al., 2004; Abourbeh et al., 2007); ML05 and PEG6-ML05 (Dissoki et al., 2007); ML06 (Shaul et al., 2004); methoxy-ML03 and *N*-{4-[(3-Bromo-phenyl)amino]-quinazoline-6-yl}-2-methoxyacetamide (“methoxy-Br-ML03”; Mishani et al., 2005); and ML10 (Mishani et al., 2008; **Figure 1**). The selected compounds represent those with high vs. low EGFR binding potency (e.g., IC50 = 0.05–5 nM, 10 nM, 30–45 nM, and 252 nM for ML06, ML04/ML05, PEG6-ML05, and methoxy-ML03, respectively, in A431 cell lysates; Shaul et al., 2004; Mishani et al., 2005; Dissoki et al., 2007) and various degrees of lipophilicity (e.g., Log *P* 3.9 for ML04, 4.12 for ML05, and 4.75 for PEG6-ML05; Dissoki et al., 2007).

## MATERIALS AND METHODS

### MATERIALS

The EGFRIs used in these experiments were kindly provided by Prof. Eyal Mishani (Department of Medical Biophysics and Nuclear Medicine, Hadassah-Hebrew University, Jerusalem, Israel). Gefitinib, the positive control, was purchased from Tocris Bioscience (Bristol, UK). BODIPY-prazosin was from Molecular Probes (Grand Island, NY, USA). The bicinchoninic acid (BCA) assay reagent kit was from Pierce (Rockford, IL, USA; Thermo Scientific). Skim milk was from Difco BD (Le Pont de Claix, France). Nitrocellulose membranes were from Whatman GmbH (Dassel, Germany). Cell culture reagents were from Biological Industries (Beit Haemek, Israel). All the other reagents were from Sigma–Aldrich (Rehovot, Israel).

### CELL CULTURES

Madin-Darbey canine kidney (MDCK) cells transfected with pcDNA empty vector (MDCK-pcDNA3; MDCK-CT) and cDNA coding for wild-type BCRP (MDCK-BCRP) cells were a generous gift from Prof. Qingcheng Mao (University of Washington, Seattle, WA, USA). The human lung carcinoma cell lines A549 and HCC827 cells were provided by Prof. Eyal Mishani.

Madin-Darbey canine kidney vector (MDCK-pcDNA3) and MDCK-BCRP cells were grown in Eagle’s minimum essential medium (MEM) supplemented with 10% fetal bovine serum, 2 mM L-glutamine, 100 units/mL penicillin, and 100 μg/mL streptomycin, at 37∘C and 5% CO_2_-humidified incubator. The BCRP positive cells were selected by supplementation of 0.05 mg/mL gentamicin to the growth medium. The adherent cells, continuously cultivated in 10 cm^2^ cell culture plates, were grown to nearly 80–90% confluence before they were harvested by trypsin-EDTA 0.25% solution. A549 were grown in Ham’s F12 growth medium and HCC827 were grown in RPMI-1640 growth medium. The cells were treated as described above.

### ATPase ASSAY

ATPase activity was evaluated using PREDEASY kits (SOLVO Biotechnology, Szeged, Hungary), according to the manufacturer’s instructions.

### WESTERN BLOT ANALYSIS

Western blot analysis was conducted as described before (Portnoy et al., 2012). Briefly, whole cell lysates were resuspended in 200 μl ice-cold lysis buffer and were shaken for 1 h at 4∘C. Then, the lysate was centrifuged at 15,100 *g* for 15 min at 4∘C. Protein concentrations were quantified by the BCA protein assay reagent kit according to the manufacturer’s instructions. Samples were run on a graded gel composed of a lower 10% separating gel layer and an upper 5% stacking gel layer. Gels were electrotransferred to nitrocellulose membranes and membranes were blocked in blocking buffer for 1 h at room temperature with gentle shaking, then washed three times with tris-buffered saline with tween 20 (TBST) for 5 min. Membranes were probed overnight at 4∘C with BXP-21 BCRP antibody at 1:250 and anti β-actin 1:2500. The membranes were washed three times with TBST for 10 min at room temperature, then incubated with horse radish peroxidase (HRP)-conjugated goat anti-rabbit secondary antibodies or goat anti-mouse IgG at 1:10000 for 1 h at room temperature. Following incubation, membranes were developed by enhanced chemiluminescence (ECL) detection and exposed to FUJI Medical Super RX X-ray films (Fujifilms, Tokyo, Japan).

### ACCUMULATION ASSAYS

Accumulation assays with fluorescent substrates of eﬄux transporter are commonly utilized to determine the inhibitory effect of a test compound (Brouwer et al., 2013). In this study, MDCK-CT and MDCK-BCRP cells were seeded at density of 20 × 10^4^ cells/well in 24 well plates. Experiments were performed two days after achieving 100% confluent monolayers. Prior to the experiment, the medium was removed and cells were incubated for 1 h with one of the test compounds dissolved in DMEM with 5 mM 4-(2-Hydroxyethyl)-1-piperazineethanesulfonic acid (HEPES), pH 7.4. In the accumulation phase cells were co-incubated with 500 nM BODIPY-prazosin (a BCRP/*P*-gp substrate; Dey et al., 1997; Ni et al., 2010) and one of the test compounds dissolved in DMEM with 5 mM HEPES. After 1 h, the cells were washed three times with ice-cold PBS. Intracellular fluorescence intensity of BODIPY-prazosin was measured within 1 h by a plate reader (Synergy HT, BioTek, Winooski, VT, USA) with excitation wavelength 485 nm and emission wavelength 528 nm.

### MTT CELL PROLIFERATION ASSAYS

The assay was performed as described earlier (Rajendra et al., 2003) with minor modifications. Briefly, Cells were seeded at density of 80 × 10^3^ cells per well in 24 well plates. On the following day, the cells were incubated (in hexaplicates) with 0.2–102.4 μM of the tested EGFRIs or with the vehicle in the growth medium for 72 h. Then, the medium was removed and 300 μl of 0.5 mg/mL MTT solution in Hank’s Balanced Salt Solution (HBSS) was added to each well. After 2 h incubation at 37∘C, the MTT solution was removed and the cells were washed three times with HBSS, then the culture plates were incubated with 500 μl dimethyl sulphoxide (DMSO) for 15 min at room temperature. The absorption was detected at 540 nm using the Synergy plate reader. The proliferation of cells from MTT studies was expressed as a percent of control, DMSO-treated cells of the same type. An inhibitory sigmoidal model was fitted to the experimental data using Phoenix WinNonlin 6.3 (Pharsight, Mountain View, CA, USA).

### STATISTICAL ANALYSIS

The Kruskal–Wallis tests was used to determine the statistical significance of differences (*p* < 0.05) between experimental groups (InStat; GraphPad, La Jolla, CA, USA). Data are presented as means ± SD. Unless otherwise stated, each treatment was applied in triplicates in two separate studies.

## RESULTS

### INTERACTIONS OF THE EGFRIs WITH BCRP ATPase

**FIGURE 2:**
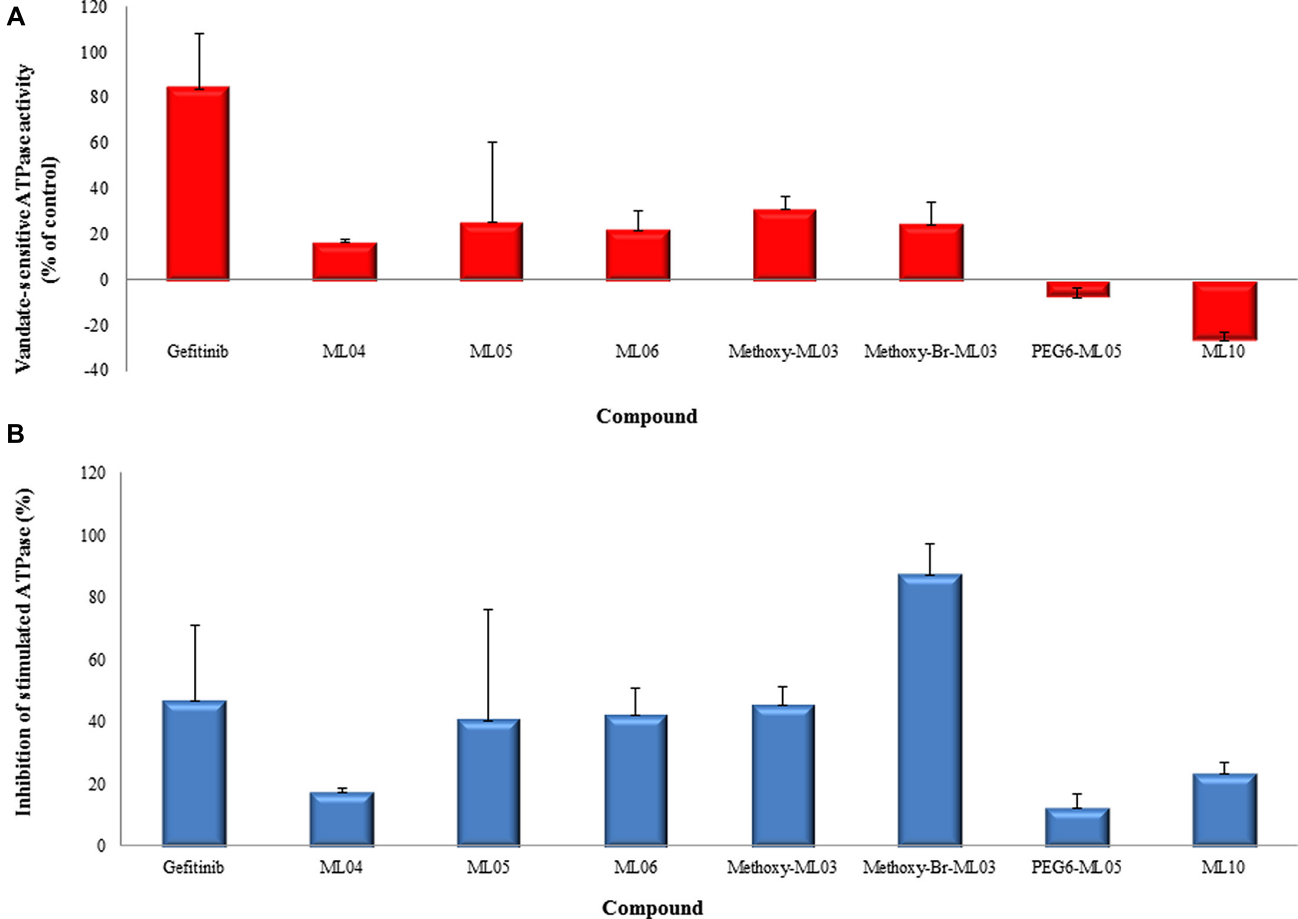
**Interactions of EGFRIs with BCRP ATPase. (A)** Activation of BCRP-ATPase in Sf9 membrane preparations containing BCRP. Membranes were incubated with ATP and the tested compounds at 400 nM in the presence and absence of sodium orthovanadate. **(B)** Inhibition of sulfasalazine-stimulated BCRP-ATPase. The effect of EGFRIs was evaluated in sulfasalazine (10 μM)-stimulated membranes. Data are presented as means ± SD of the vanadate-sensitive ATPase activity from two experiments in duplicates.

**Figure 2** demonstrates the effects of the tested EGFRIs on human BCRP ATPase activity in Sf9 membrane preparations. ML04, ML05, ML06, and the two methoxy derivatives of ML03 stimulated the BCRP ATPase, although activation did not exceed 40% of that produced by gefitinib (**Figure 2A**). PEG-6-ML05 and ML10 had no activating effect on ATPase activity. With the exception of methoxy-Br-ML03, all the compounds inhibited BCRP ATPase to various extent (**Figure 2B**).

### EFFECTS OF BCRP OVEREXPRESSION ON THE ANTIPROLIFERATIVE ACTIVITY OF EGFRIs

**FIGURE 3:**
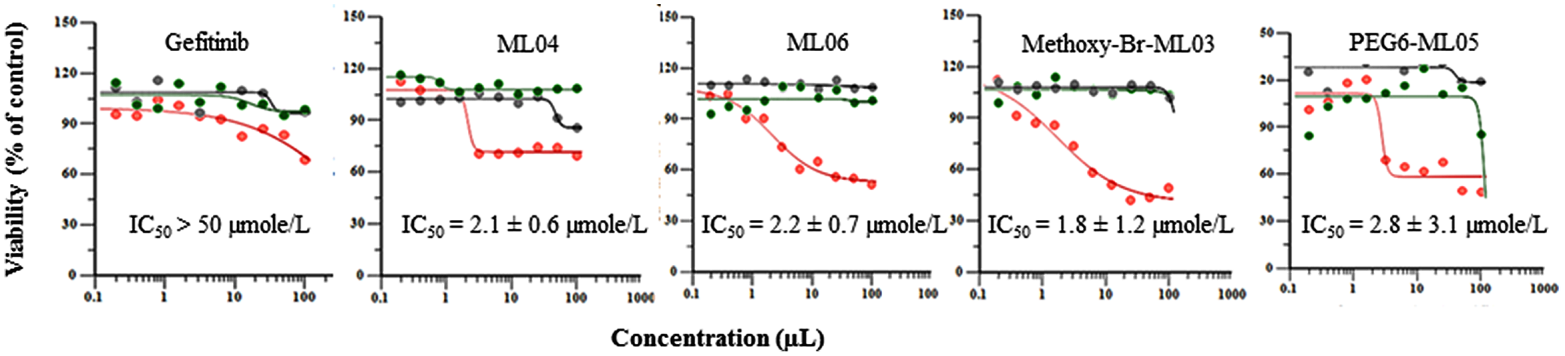
**Effects of BCRP overexpression on the cytotoxicity of EGFRIs by the MTT assay.** Stable MDCKII transfectants of vector alone or vectors expressing wild-type BCRP were incubated for 72 h with various concentrations of the indicated compounds. Results are shown as experimental findings (circles) and predicted model (line) in MDCK-CT cells (red), MDCK-BCRP cells (black), and MDCK-BCRP cells treated with 20 μM verapamil, to block endogenous *P*-gp activity (green). Data represent the means ± SD of six replicates.

Madin-Darbey canine kidney II stable transfectants were used to assess the effect of human BCRP overexpression on the antiproliferative activities the EGFRIs (**Figure 3**). In a separate control group, 20 μM verapamil was used to block the effect of the endogenous, canine *P*-gp present in MDCK cells (Poller et al., 2011). Inhibition of cell proliferation by ML04, ML06, methoxy-Br-ML03, and PEG6-ML05 occurred at milimolar concentrations, and overexpression of BCRP conferred resistance to this effect (**Figure 3**). The other tested compounds did not affect the proliferation of MDCK-CT and MDCK-BCRP cells at concentrations up to 50 μM (data not shown).

### EFFECT OF THE EGFRIs ON BODYPY-prazosin ACCUMULATION

**FIGURE 4:**
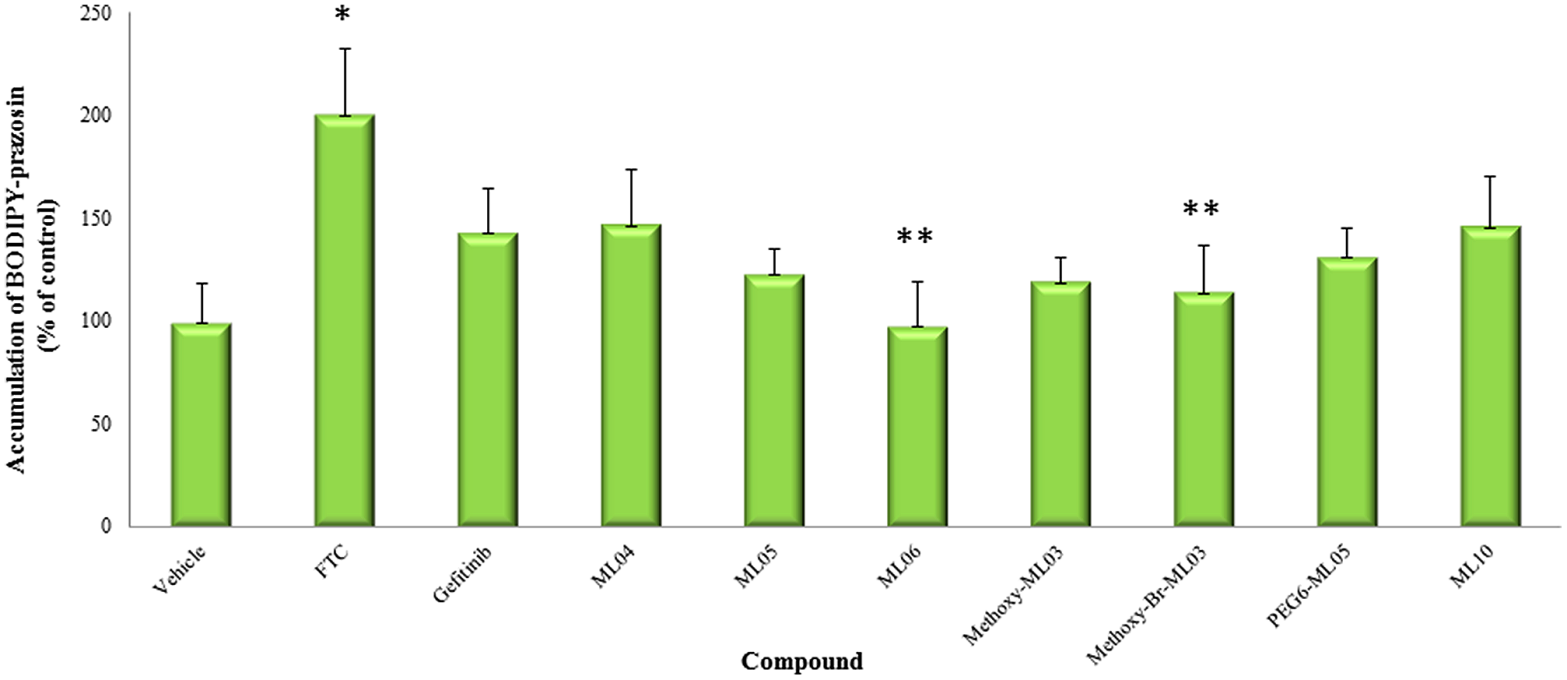
**Effect of EGFRIs on BODIPY-prazosin accumulation.** Cell protein concentration-normalized BODIPY prazosin fluorescence in MDCK-BCRP cells were evaluated in the presence and the absence of 400 nM of the tested compounds following 1 h incubation. Results are presented as means ± SD. *Significantly different from vehicle-treated cells, *P* < 0.01; **Significantly different from FTC-treated cells, *P* < 0.01.

Although some of the tested compounds partially inhibited BCRP ATPase activity, this was not reflected in the cellular assay (**Figure 4**). Only the positive control, FTC, significantly (*p* < 0.01) enhanced BODIPY-prazosin accumulation in MDCK-BCRP cells. None of the compounds significantly affected the fluorescent signal of MDCK-CT cells (data not shown).

### RELATIVE BCRP EXPRESSION IN TUMOR CELLS

**FIGURE 5:**
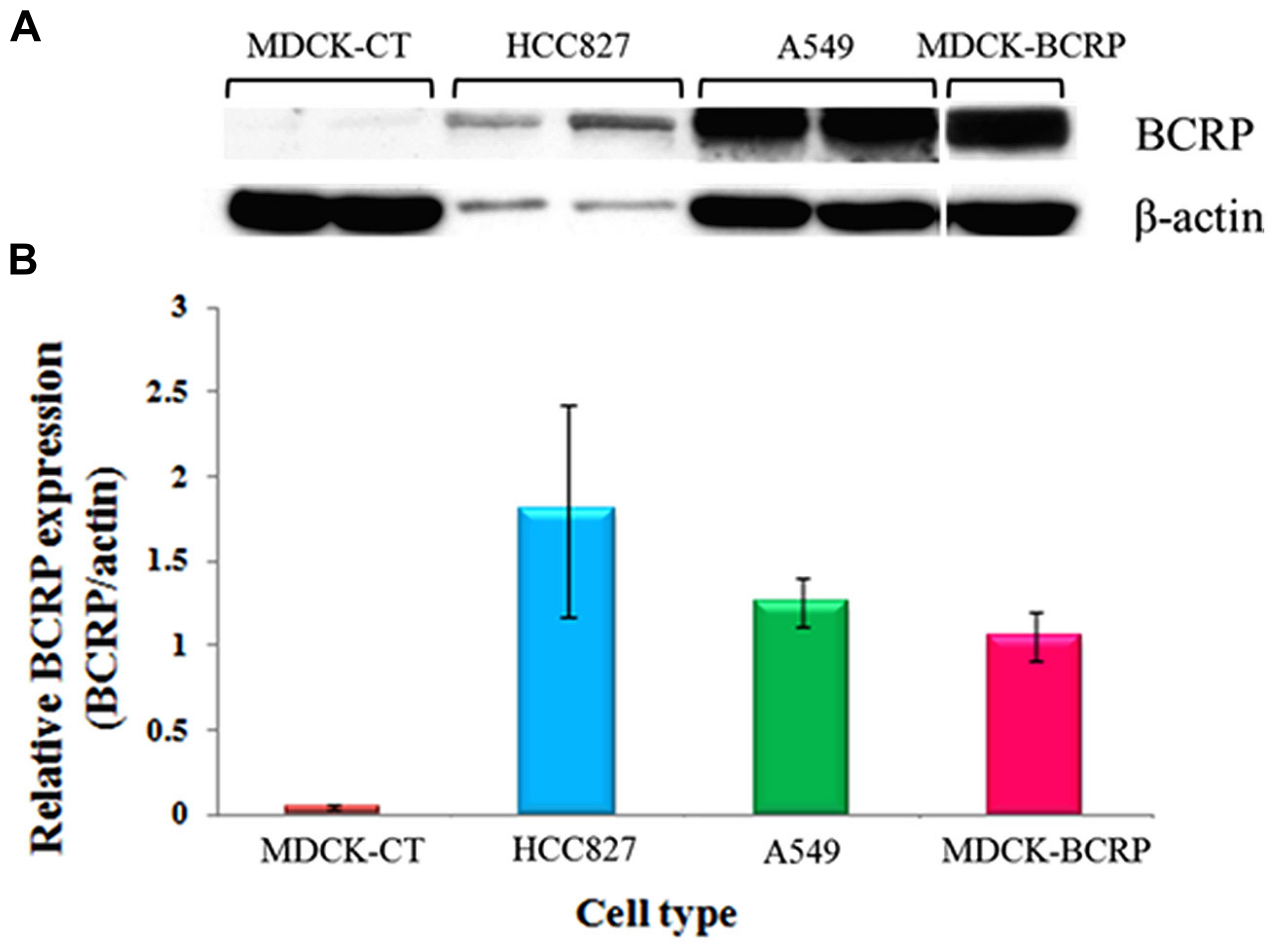
**BCRP protein expression in HCC827 and A549 cells. (A)** Representative image demonstrating the intensity of BCRP expression. **(B)** Relative β-actin-normalized BCRP expression. MDCK-BCRP and MDCK-CT cells were used as positive and negative controls, respectively. *n* = 4 for each cell type.

HCC827 and A549 cells have been previously used for *in vivo* imaging of EGFR expression and showed variability in tracer accumulation. The expression of both *P*-gp and BCRP in A549 cells has been reported before (Scharenberg et al., 2002). Our analysis confirmed this finding and further demonstrated BCRP expression in HCC827 cells. In both cell lines, the extent of BCRP expression was comparable to that in MDCK-BCRP cells (**Figure 5**).

## DISCUSSION

The increasing need for personalized treatment with EGFRIs promoted the development of novel PET EGFR biomarkers. Indeed, encouraging results have been obtained through preclinical studies with both competitive and non-competitive EGFRIs. Yet, the determinants of probe distribution into tumors are not fully understood, because the specificity of tumor signal intensity may be affected by both the amount of the probe that distributes into tumor cells and its binding to its cellular targets. This study evaluated the interaction of several novel EGFR probes with BCRP, an ABC transporter that has been previously implicated in EGFRIs eﬄux (Ni et al., 2010). The BCRP ATPase and the BCRP inhibition assays were conducted at submicromolar concentrations, to reflect clinically relevant, unbound EGFRIs plasma levels. This is particularly the case in PET imaging studies, in which ligand microdoses may be used.

### EGFRIs INTERACTIONS WITH BCRP

Five of the tested compounds, namely ML04, ML05, ML06, methoxy-ML03, and methoxy-Br-ML03, activated BCRP ATPase at submicromolar concentrations, suggesting that these compounds are transported by BCRP. The values obtained with ML10 and PEG6-ML05 could result from inhibition of basal Pi release (manufacturer’s brochure). A partial overlap observed between ATPase stimulation and inhibition indicated that that the evaluated compounds may function as competitive inhibitors which are also transported substrates (Eadie et al., 2014).

Compared to the relatively low concentrations which stimulated the BCRP ATPase, greater EGFRIs concentrations were required to affect the proliferation of MDCK-CT cells. This reflects both the distributional barrier into cells and the degree sensitivity to the toxic effects of EGFRIs of MDCK cells, which have been shown to express EGFR (Pick and Wiese, 2012). The mechanisms of EGFRIs effects on MDCK cell proliferation are currently unknown, although a recent study reported on the role of EGFR in mitotic spindle orientation in this cell line (Bañón-Rodríguez et al., 2014).

At 400 nM, none of the tested compounds increased the accumulation of BODYPY-prazosin in MDCK-BCRP cells. It is unlikely that the low plasma concentrations of EGFRIs obtained upon their administration as microdoses in PET imaging studies will be involved in pharmacokinetic interactions with *P*-gp/BCRP substrates. Yet, BCRP inhibition-based interactions at greater EGFRIs concentrations cannot be ruled out.

Our study could have been strengthened by using the eﬄux ratio assay to distinguish between inhibitors and substrates and to gain a better understanding of the mechanisms that control the distribution of the compounds of interest into cells. We used the ATPase assay due to its simplicity, and subsequently evaluated the cellular effects of the TKIs as a marker of their BCRP-mediated transport. However, future characterization of these compounds will involve the use of bidirectional transport assays.

Due to the relatively small number of molecules evaluated in our study, it is difficult to assess the impact of structural modifications on the molecule interactions with BCRP. Furthermore, BCRP is predicted to have multiple binding sites with overlapping specificities (Ni et al., 2010). Thus, the interactions of substrates with the transporter cannot be precisely identified. It has been suggested that substrate recognition is based on global physiochemical properties, such as carbon size length and lipophilicity, although the importance of log *P* has been debated (Ni et al., 2010; Szafraniec et al., 2014). A more sophisticated structure activity relationship study with SN-38 and its analogs suggested that one amine bound to one carbon of a heterocyclic ring, fused heterocyclic rings, and two substituents on a carbocyclic ring of the fused heterocyclic are important factors in the molecule’s interaction with BCRP (Saito et al., 2006). That study also predicted a strong interaction of gefitinib with BCRP based on these features. Chemical modifications such as those aimed to achieve irreversible EGFR binding may reduce the potency of the EGFRI in binding BCRP. This is supported by the results obtained in the ATPase activation assay (**Figure 2A**). For example, PEGylation of ML04 and ML05, aimed at reducing their log *P* with resultant lower non-specific binding (Dissoki et al., 2007; Mishani et al., 2008; Mishani and Hagooly, 2009), could have affected the recognition of these compounds by both BCRP (**Figure 2**) and *P*-gp. Nevertheless, no trend was observed with regard to the relationships between EGFR inhibition potency or lipophilicity of the compound and its interactions with BCRP.

### TUMOR CELLS EXPRESSION OF *P*-gp AND BCRP

Preclinical PET and biodistribution studies in animal bearing tumor xenografts demonstrated high and sustained uptake of ^11^C-erlotinib in HCC827, which harbor an activating mutation in exon 19, compared to A549 and NC1358 tumors (Memon et al., 2009). In order to better understand the impact of BCRP on the distribution of the studied compounds in experimental tumor models, we also examined BCRP expression in HCC827 and A549 cells. In both cell lines, BCRP expression was comparable to that observed in the positive control, MDCK-BCRP. Therefore, it appears that the irreversible binding of the EGFRI to its intracellular target could potentially contribute to overcoming the distributional restriction, at least in part, due to relatively longer residence within cells.

## CONCLUSION

Our data suggest that some of the EGFRIs evaluated in this study interact with BCRP. Whether the compound functions as a transported substrate or an inhibitor may depend on its concentrations, the cell type and the experimental or clinical setting. Accordingly, low radioligand concentrations, in contrast to concentrations aimed to clinically inhibit EGFRs, could have resulted in a more pronounced BCRP/*P*-gp eﬄux and may be one explanation for the failure of some successful targeted EGFRIs as tracer imaging agents (Mishani et al., 2008). Therefore, using a mixture of radiolabeled tracer and a “cold” compound to reflect clinically relevant concentration might improve the outcome of the preclinical imaging studies.

The relative role of transporter-mediated eﬄux vs. target binding in probe accumulation within tumors is currently unknown. To address this issue, the activity of novel compounds developed as EGFR probes can be tested *in vitro* in the presence and the absence of ABC transporters inhibitors. Likewise, given the expression of BCRP and *P*-gp in cell lines commonly used for tumor grafting in small animals, *in vivo* studies conducted in the presence and the absence of transporter inhibitors may help distinguish between pharmacokinetic and pharmacodynamic contribution to the radioactivity signal within tumors. Those radioligands which are found to be good *P*-gp/BCRP substrates and do not avidly bind EGFRs may be utilized for *in vivo* imaging studies of *P*-gp/BCRP functional activity at the blood brain barrier. Importantly, further interactions with other eﬄux transporters (multidrug resistance-associated proteins; MRPs) and relevant uptake transporters should be investigated in order to better understand the biodistribution of these probes.

## Conflict of Interest Statement

The authors declare that the research was conducted in the absence of any commercial or financial relationships that could be construed as a potential conflict of interest.

## ABBREVIATIONS

ABC: adenosine triphosphate binding cassette
BCRP: breast cancer resistance protein
EGFR: epidermal growth factor receptor
EGFRI: epidermal growth factor receptor kinase inhibitor
FTC: fumitremorgin C
PET: positron emission tomography
P-gp: P-glycoprotein
TKI: tyrosine kinase inhibitor
